# Adenomyosis: an underacknowledged cause of abnormal uterine bleeding and pelvic pain

**DOI:** 10.1016/j.ebiom.2025.105656

**Published:** 2025-03-12

**Authors:** eBioMedicine

Up to a third of women will experience abnormal uterine bleeding during their life. One cause of this is adenomyosis, a gynaecological condition characterised by the infiltration of endometrial tissue into the myometrium layer of the uterus. Although the condition can be asymptomatic, many women with adenomyosis experience menorrhagia, dysmenorrhea, chronic pelvic pain, and reduced fertility. Despite the substantial burden on wellbeing, there are no approved therapies for adenomyosis, and research lags behind even that of other gynaecological conditions, such as endometriosis and uterine leiomyoma, with which it is often comorbid.

In a scoping review published in *BMJ Open* in January, 2025, the lived experience and diagnostic journey of women with adenomyosis was examined. The review, which included six eligible studies, highlighted not only the profound physical and mental health impacts of living with adenomyosis, but also the personal effects on relationships, employment opportunities, and finances. Many women reported feeling dismissed by health-care providers. The authors conclude that “a cultural shift is required” to address this perceived lack of empathy and to improve diagnosis and management options going forward. This conclusion echoes the findings of a wider report on painful reproductive health conditions published in December, 2024 by the Women and Equalities UK Parliament Committee. The report condemned the inadequate options for women in the UK with these gynaecological conditions and the lack of awareness and knowledge that has seen women have their pain dismissed by health-care professionals and normalised by society. Improved access to diagnostic referral pathways to investigate the underlying causes of abnormal uterine bleeding and chronic pelvic pain is urgently needed—especially since conditions such as endometriosis and adenomyosis can be progressive.

Definitive diagnosis of adenomyosis has typically been achieved through post-hysterectomy histological examination, which has led to the incorrect observation that adenomyosis is a disease of older, multiparous women, who are more likely to undergo a hysterectomy. Increasing recognition of pathological features on imaging, namely transvaginal ultrasound (TVUS) and MRI, has enabled diagnosis without the need for surgery. In a systematic review published in *F&S Reviews*, adenomyosis was detected in 16·9% of symptomatic adolescents (younger than 20 years) and 29·7% of symptomatic young women (age 25 years and younger). Reports of prevalence vary based on the sampled population, method of identification, and diagnostic criteria used. For example, a 2021 retrospective study including 307 surgical patients, published in *Annals of Medicine and Surgery**,* found adenomyosis in 42% of patients who had a hysterectomy, with this figure rising to 59% in the group who sought a hysterectomy for abnormal uterine bleeding. An earlier prospective TVUS study, published in *Human Reproduction**,* examined a consecutive series of 985 patients attending a gynaecological clinic and estimated the presence of adenomyosis in 20% of participants. An accurate prevalence for adenomyosis in the general population, therefore, remains unclear.

In 2015, the Morphological Uterus Sonographic Assessment (MUSA) group published a consensus statement in *Ultrasound in Obstetrics and Gynecology*, detailing sonographic features of the myometrium, including proposed criteria for adenomyosis identification. This statement was updated in a Delphi study, published in 2022, in which experts recommended the use of 3D ultrasound to optimise visualisation of the uterine junctional zone. Sonographic features, such as myometrial cysts and hyperechogenic islands, directly indicate the presence of endometrial tissue in the myometrium; other features, such as an irregular junctional zone, were considered indirect characteristics of adenomyosis on ultrasound. However, despite these revised guidelines, identification of adenomyosis on imaging remains challenging in many clinics. A January, 2025 publication in the *Journal of Clinical Medicine* examined the reproducibility of the MUSA guidelines and reported only modest inter-observer and intra-observer agreement using the direct ultrasound features to report suspected adenomyosis. Similarly, a June, 2023 *Diagnostics* study found that, although inter-observer agreement was higher when using TVUS compared with MRI, measurement of the junctional zone by 3D ultrasound was unreliable. Advances in artificial intelligence and machine learning might prove valuable in aiding the classification of adenomyosis on imaging, as has been previously shown for cases such as focal liver lesions, breast cancer, and bone lesions. Combining imaging features with multiomic data is a promising avenue of research, and a multicentre trial is currently ongoing to assess if this can improve early diagnosis and personalised treatment recommendations for patients with endometriosis and adenomyosis (NCT06572852).

The complexity of diagnosis is just one issue contributing to the inadequate medical care of women with adenomyosis. The lack of treatment guidelines and the limited availability of high-quality evidence has impaired therapeutic management and prevented standardisation of care. Hysterectomy remains the only definitive treatment option. Medical management has largely focused on alleviating the symptoms of dysmenorrhea and dyspareunia, and can involve the use of nonsteroidal anti-inflammatory drugs, tranexamic acid, progestins (eg, levonorgestrel-releasing intrauterine system and dienogest), and gonadotropin-releasing hormone (GnRH) agonists. Thermal ablation has been used for the treatment of adenomyosis-related menorrhagia in women who do not wish to preserve fertility. In a retrospective study of 120 women, published in the *European Journal of Obstetrics & Gynecology and Reproductive Biology* in January, 2025, the efficacy of high-intensity focused ultrasound (HIFU) combined with endometrial thermal balloon ablation (TBEA) was examined. Concurrent HIFU and TBEA treatment was found to consistently lower menorrhagia in follow-up at 6, 9, and 12 months compared with HIFU alone, and patients in this group reported higher quality of life scores. However, longer term follow-up is required. A network meta-analysis aiming to identify the best treatment for adenomyosis was published in the *Journal of Ultrasound in Medicine* in December, 2024, and reported that HIFU combined with GnRH agonists and progestin was more effective in decreasing dysmenorrhea scores than other combined therapies.

For women who do wish to preserve fertility, treatment options are even more limited. It has been hypothesised that selectively inhibiting angiogenesis might represent a novel therapeutic approach that offers the possibility of fertility preservation. Abnormal vascularisation and increased expression of pro-angiogenic factors, such as vascular endothelial growth factor (VEGF**)**, have been observed in adenomyotic endometrial tissue. To this end, Harmsen and colleagues reported angiogenesis inhibition in a tamoxifen-induced mouse model of adenomyosis published in *Angiogenesis* in January, 2025. Mice received either a low dose of axitinib (a VEGF-R2 inhibitor), a high dose of axitinib, or a vehicle-only placebo. High grade adenomyosis was observed less in the high-dose (55·9%) and low-dose (48·0%) treated mice compared with the placebo group (79·4%). Downregulation of VEGF-R1 and VEGF-R2 was observed in the low-dose axitinib group, and downregulation of VEGF-R13–, VEGF-A, and VEGF-B was achieved in the high-dose group. Axitinib is approved for use in humans and is used in the treatment of kidney cancer, albeit with adverse side effects. The drug is currently in a phase 2 trial for the treatment of metastatic endometrial cancer in combination with avelumab (NCT02912572). It is crucial that future studies evaluate the plausibility of long-term applications of drugs, such as selective angiogenesis inhibitors in adenomyosis. Although such proof-of-concept studies are a long way from clinical application, they nevertheless offer hope that pharmaceutical management of adenomyosis progression might one day be possible.

Too many women are living with the pain of understudied and underdiagnosed gynaecological conditions. At *eBioMedicine,* we look to a future where the prevalence and impact of severe dysmenorrhea and menorrhagia are recognised and where improved diagnostic tools and options for appropriate medical care exist. We welcome research submissions that aim to bring this future forward: not only for adenomyosis, but for all painful gynaecological health conditions.

